# From Genome to Geroscience: How DNA Damage Shapes Systemic Decline

**DOI:** 10.1002/bies.70051

**Published:** 2025-08-10

**Authors:** Athanasios Siametis, George A. Garinis

**Affiliations:** ^1^ Institute of Molecular Biology and Biotechnology (IMBB) Foundation for Research and Technology‐Hellas Heraklion Crete Greece; ^2^ Department of Biology University of Crete Heraklion Crete Greece

## Abstract

Persistent genomic instability compromises cellular viability while also triggers non‐cell‐autonomous responses that drive dysfunction across tissues, contributing to aging. Recent evidence suggests that DNA damage activates secretory programs, including the release of inflammatory cytokines, damage‐associated molecular patterns, and extracellular vesicles, that reshape immune homeostasis, stem cell function, and metabolic balance. Although these responses may initially support tissue integrity and organismal survival, their chronic activation has been associated with tissue degenerative changes and systemic decline. Here, we discuss how nuclear DNA damage responses trigger the activation of cytoplasmic sensing pathways, promote secretory phenotypes, and affect organismal physiology. Targeting DNA damage‐driven mechanisms may help buffer harmful systemic responses while preserving regeneration and immune surveillance, offering new ways to delay aging‐related decline.

## Graphical abstract

Persistent DNA damage triggers systemic secretory responses including damage‐associated molecular patterns (DAMPs), the senescence‐associated secretory phenotype (SASP), and extracellular vesicles (EVs). This non‐cell‐autonomous signaling disrupts intercellular communication, driving organism‐wide dysfunction and aging, and offering new therapeutic targets.

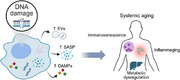

## 1 Introduction

Genomic instability causally contributes to aging and the premature onset of a wide range of age‐related diseases [1, 2, 3]. DNA lesions, arising from toxic metabolic byproducts, transcription, DNA replication, or environmental insults, trigger a multilayered DNA damage response (DDR) that involves an arsenal of DNA damage sensors and signal transducers to detect the myriad structural DNA modifications as well as a series of partially overlapping, highly conserved DNA repair mechanisms [4] aimed at preserving genomic integrity. Although the canonical DDR acts within the damaged cell, accumulating evidence suggests that genomic instability can also trigger systemic responses. These responses extend well beyond the nucleus to impinge on neighboring cells and distant tissues. For instance, the DDR may trigger the release of damage‐associated molecular patterns (DAMPs), inflammatory cytokines, and extracellular vesicles (EVs). These secreted factors act through autocrine, paracrine, or endocrine routes, modulating the tissue microenvironment, organ physiology, and organismal survival over time [5, 6]. In the short‐term, DNA damage‐driven secretory mechanisms may prove beneficial for the survival of cells and the organism. However, in the long run, persistent activation of these responses leads to chronic inflammation, metabolic decline, and the onset of degenerative diseases [7, 8, 9, 10]. Here, we examine how DNA damage, through its cytoplasmic signaling and secretory outputs, drives systemic responses that contribute to tissue dysfunction and organismal aging. We place particular emphasis on emerging strategies to modulate damage‐induced intercellular communication to mitigate age‐related decline.

## 2 Canonical Nuclear Responses to DNA Damage

DNA is inherently vulnerable to damage and mutations, both of which can compromise cellular function and organismal health. Although DNA lesions are structural disruptions that can impede cell processes, mutations cause permanent, inheritable changes to the genetic code. Interestingly, some DNA breaks are essential, such as those occurring during meiosis or immune cell development. However, most breaks arise spontaneously or from environmental genotoxins [11, 12], contributing to cellular malfunction, aging, and disease, including cancer. To address the constant threat of genomic damage, cells have developed a highly coordinated and tightly regulated defense system known as the DDR. This network includes specialized sensors and signaling molecules [13, 14] that detect a wide range of DNA lesions and relay the damage signal throughout the cell. The DDR initiates a cascade of events, including activation of cell cycle checkpoints, chromatin remodeling, and the recruitment of a series of partially overlapping DNA repair pathways. For example, base excision repair (BER) corrects small base lesions from oxidation, deamination, or alkylation; mismatch repair (MMR) resolves replication errors; and nucleotide excision repair (NER) eliminates bulky DNA adducts caused by UV radiation or chemical exposure. More severe damage, such as double‐strand breaks (DSBs), is repaired either through non‐homologous end joining (NHEJ), which directly ligates the DNA ends, or homologous recombination (HR), which uses a homologous sequence as a template. These repair pathways address diverse lesions. Notably, damage that blocks RNA polymerase II (RNAPII) during transcription specifically activates transcription‐coupled repair mechanisms as part of the overall DDR [15]. Although highly efficient, DNA repair mechanisms are ultimately unable to contend indefinitely with the full spectrum of endogenous DNA lesions affecting the mammalian genome. Consequently, multicellular organisms must endure long‐lasting DNA damage while simultaneously maintaining physiological integrity and defending against the early emergence of disease, particularly cancer. When repair is insufficient and damage persists, cells undergo apoptosis that eliminates severely damaged cells to prevent malignant transformation or senescence that arrests cells in a stable, non‐proliferative state [6, 12]. Importantly, the DDR does not function in isolation. It integrates with metabolic regulation, redox balance, stress signaling, and resource management to guide cell fate decisions under genotoxic stress, while limiting the harmful effects that damaged or dysfunctional cells may exert on the surrounding environment.

## 3 From Nuclear Damage to Cytoplasmic Responses

### 3.1 Nuclear DNA Damage‐Driven Cytoplasmic DNA Release

**FIGURE 1:**
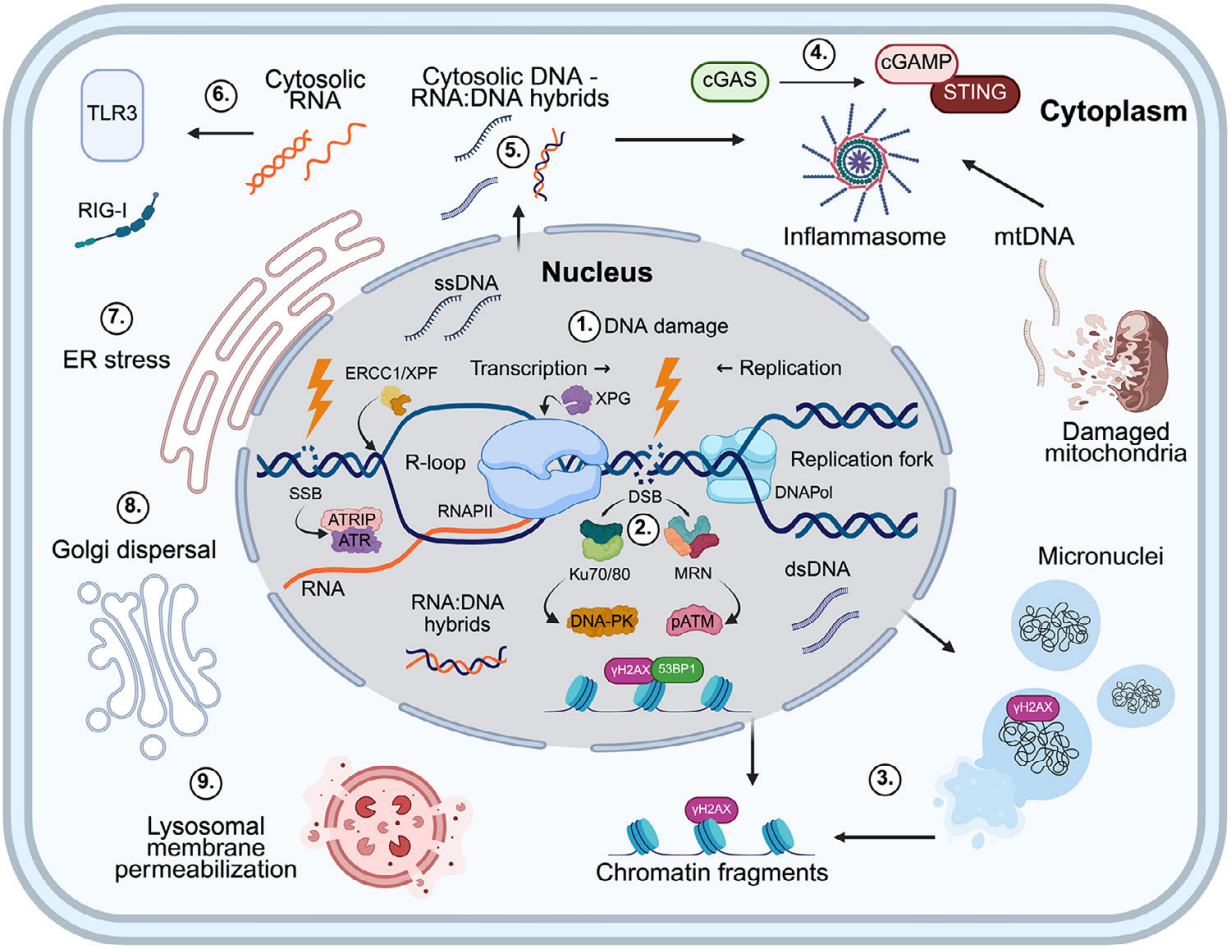
Nuclear DNA damage triggers cytoplasmic nucleic acid release, innate immune sensing, and organelle stress. Genotoxic insults induce DNA lesions, including single‐ and double‐strand breaks (SSBs and DSBs), and interfere with transcription and replication, leading to R‐loop formation, replication fork stalling, and transcription–replication collisions (1). Unresolved damage activates canonical DNA damage response (DDR) signaling via ATM, ATR, DNA‐PK, and their associated repair complexes (e.g., MRN, Ku70/80) (2). Persistent damage promotes the accumulation of nuclear‐derived cytoplasmic DNA species, such as chromatin fragments and DNA resulting from micronuclei rupture, often marked by DNA damage markers (e.g., γH2AX) (3). These cytoplasmic DNAs, as well as mitochondrial DNA (mtDNA) released from dysfunctional mitochondria, are recognized by innate immune sensors such as cGAS, which activates STING and triggers downstream inflammatory signaling, while longer or oxidized DNA fragments engage inflammasomes like AIM2 and NLRP3 (4). R‐loops, along with DNA lesions that stall transcription, are processed by endonucleases like XPG, ERCC1, and XPF, producing RNA:DNA hybrids and single‐stranded DNA (ssDNA) fragments that can accumulate in the cytoplasm and activate immune sensors (5). Cytoplasmic RNA species are detected by RIG‐I and TLR3 (6). Beyond nucleic acid sensing, persistent DDR signaling perturbs endoplasmic reticulum (ER) proteostasis (7), disrupts Golgi architecture (8), and induces lysosomal membrane permeabilization (9), linking nuclear damage to organelle dysfunction and reinforcing inflammatory output. Created in BioRender. Siametis, A. (2025) https://BioRender.com/aticwwh.

**FIGURE 2:**
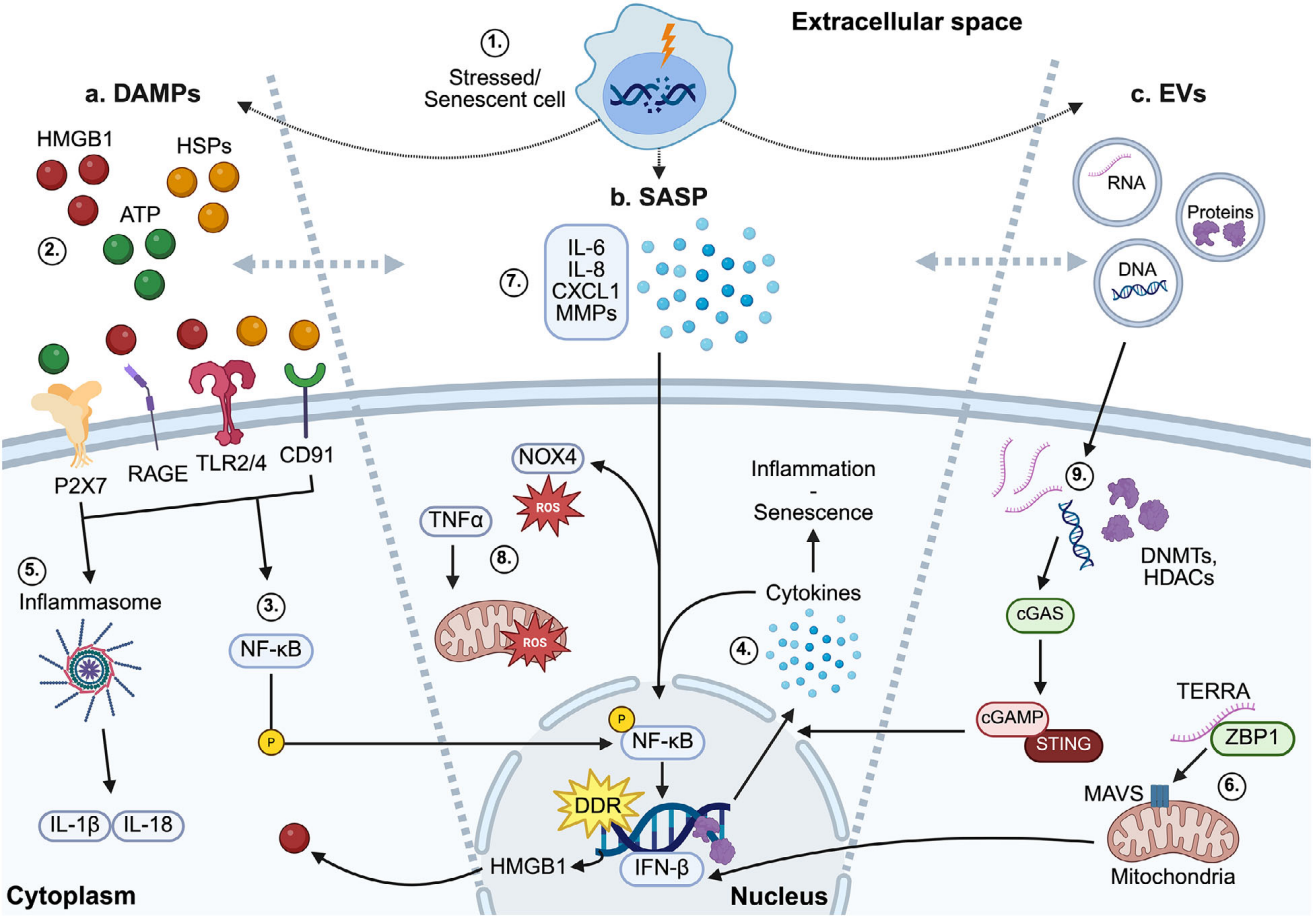
Persistent DNA damage activates secretory programs that mediate non‐cell‐autonomous stress communication. Genotoxic stress induces the release of (a) damage‐associated molecular patterns (DAMPs), (b) senescence‐associated secretory phenotype (SASP) factors, and (c) extracellular vesicles (EVs), that propagate inflammatory and senescence‐associated signals across tissue boundaries (1). DAMPs such as HMGB1, extracellular ATP, and heat shock proteins (e.g., HSP70, HSP90) (2) are released and activate innate immune receptors including TLRs, RAGE, and purinergic receptors, promoting NF‐κB activation (3), cytokine production (4), and converging on inflammasome activation (5). Mitochondrial dysfunction and NOX4 activity elevate reactive oxygen species (ROS), which amplify DNA damage and enhance proinflammatory signaling (6). Persistent DNA damage also induces SASP, marked by secretion of cytokines (e.g., IL‐6, IL‐8), chemokines (e.g., CXCL1), growth factors, and matrix metalloproteinases (MMPs), which reinforce senescence in neighboring cells and disrupt tissue structure and regeneration (7). EVs from stressed cells, enriched in DNA fragments, regulatory RNAs, and epigenetic modulators (e.g., DNMTs, HDACs) act in autocrine, paracrine, and endocrine‐like manner, activating cGAS‐STING signaling, modulating metabolism, and promoting senescence and dysfunction (8). Telomeric repeat‐containing RNA (TERRA), released under telomeric stress, triggers MAVS‐dependent Type I interferon responses (9). Together, these secretory programs contribute to immunosenescence, tissue degeneration, and systemic aging. Created in BioRender. Siametis, A. (2025) https://BioRender.com/41tqudf.

Persistent nuclear DNA damage initiates structural and transcriptional changes that promote the accumulation of self‐DNA in the cytoplasm [16] (Figure 1, 2). Unresolved genomic stress often disrupts the nuclear envelope. This process is frequently initiated by DDR‐mediated phosphorylation of lamin A/C through ATM and ATR signaling pathways [17]. This destabilizes the nuclear lamina and facilitates the formation of micronuclei (Figure 1.3). These structures are membrane‐bound compartments that encapsulate lagging chromosomes or missegregated DNA fragments during mitosis [17, 18, 19]. Due to defective lamina and nuclear pore complex assembly, micronuclei are structurally unstable and prone to rupture, releasing chromatin into the cytoplasm [20]. Micronuclear chromatin often carries DNA damage markers such as γH2AX and serves as a persistent source of cytoplasmic DNA in DNA repair‐deficient or senescent cells [21].

Cytoplasmic chromatin fragments (CCFs) represent another class of nuclear‐derived DNA observed in response to persistent genotoxic stress. Unlike micronuclei, CCFs form independently of mitosis, particularly in senescent cells. Their generation is linked to DDR signaling and heterochromatin remodeling. MRE11‐mediated DNA end resection promotes CCF formation, while 53BP1, a key factor in DSB repair, acts as a negative modulator [22]. Loss of lamin B1, via transcriptional repression and autophagic degradation, facilitates chromatin detachment from the nuclear periphery and promotes CCF release [23, 24]. CCFs are typically heterochromatic and enriched in DNA damage markers (Figure 1.3), consistent with their origin in compromised genomic regions.

DNA damage‐associated transcription stress further contributes to extranuclear DNA accumulation. During transcription elongation, DNA lesions, R‐loops, or transcription‐DNA replication collisions can stall RNAPII, threatening genome stability and promoting DNA breakage [25]. R‐loop structures, composed of RNA:DNA hybrids and a displaced single DNA strand, are particularly vulnerable to endonucleolytic cleavage by XPG and XPF structure‐specific endonucleases, generating fragments that can be released into the cytoplasm [26, 27, 28]. This process is exacerbated in cells lacking BRCA1, SETX, or RNase H2, where R‐loop resolution is impaired [29]. Transcription stress at telomeres was also recently shown to release telomeric DNA in the cytosol of cells, supporting the notion that hampering transcription causally contributes to cytoplasmic DNA burden at multiple genomic loci [30].

Retrotransposon reactivation becomes more prominent under conditions of nuclear DNA damage and epigenetic deregulation [31]. For instance, loss of silencing at LINE‐1 elements promotes their transcription and reverse transcription, generating cytoplasmic cDNA intermediates [32]. If these intermediates escape reintegration or degradation, especially in contexts where exonucleases such as TREX1 are repressed, they accumulate in the cytosol and amplify immunostimulatory nucleic acid pools [33].

Although mitochondrial DNA (mtDNA) originates independently of the nucleus, its cytoplasmic release often reflects nuclear genome instability. DDR activation is associated with elevated reactive oxygen species (ROS), PARP1 hyperactivation, and changes in NAD+/SIRT1 signaling that impair mitochondrial biogenesis and mitophagy [34, 35]. As a result, damaged mitochondria accumulate and release oxidized or fragmented mtDNA, often via BAX/BAK‐dependent membrane permeabilization [36]. In turn, this mtDNA contributes to the growing pool of cytosolic nucleic acids that accumulate during persistent nuclear stress [37, 38].

### 3.2 Cytoplasmic Sensing of Damage‐Associated Nucleic Acids

Cytoplasmic DNA generated by unresolved nuclear damage activates innate immune sensors that detect self‐nucleic acids and initiate inflammatory responses (Figure 1.4, 5). The cyclic GMP‐AMP synthase (cGAS)‐STING pathway is the best characterized among these systems. When cGAS binds double‐stranded DNA (dsDNA), it synthesizes cyclic GMP‐AMP (cGAMP), which then activates STING on the endoplasmic reticulum (ER). This activation triggers phosphorylation of TBK1, followed by IRF3 and NF‐κB activation, leading to expression of Type I interferons and proinflammatory cytokines such as IL‐6 and TNF‐α [39]. Although this response promotes the clearance of damaged cells, chronic activation, as seen in aging or DNA repair‐deficient contexts, can drive persistent tissue inflammation and degeneration [40, 41].

The structure, length, and modification status of DNA strongly influence sensor engagement. cGAS preferentially binds longer B‐form dsDNA (>45–50 bp) while oxidized or fragmented DNA may activate alternative pathways [40]. AIM2, another cytosolic sensor, recognizes long dsDNA fragments (>80 bp) and activates inflammasome signaling via ASC and caspase‐1 recruitment, leading to IL‐1β maturation [42, 43]. Z‐DNA‐binding protein ZBP1 detects Z‐DNA, expanding the spectrum of cytoplasmic DNA surveillance [44]. Additional sensors, such as IFI16 and DDX41, function upstream of STING or independently, suggesting a degree of redundancy and context‐dependent crosstalk [45, 46]. Recent findings show that even chromatin‐associated or nucleosome‐bound DNA can be sensed under certain stress conditions. Although nucleosomal wrapping limits cGAS activity, exonucleases like TREX1 can process micronuclear or CCF‐derived DNA exposing fragments for immune sensing [47].

DNA damage‐associated transcription stress also contributes to cytoplasmic nucleic acid sensing. R‐loops formed during transcription can be cleaved by endonucleases such as XPG and XPF, releasing RNA:DNA hybrids or single‐stranded DNA into the cytoplasm [26, 27, 28]. These hybrid fragments can activate cGAS and RNA‐sensing pattern recognition receptors (PRRs) like RIG‐I and TLR3 [40, 41] (Figure 1.6). Although a dedicated sensor for RNA:DNA hybrids has not been identified, ZBP1 can recognize such hybrids in the Z‐conformation [48].

DNA quality further influences the immune response. Oxidized mtDNA and damaged nuclear fragments often activate inflammasomes (e.g., NLRP3, AIM2), while intact dsDNA favors cGAS–STING signaling [37, 49]. The subcellular location, timing of DNA release, and sensor composition, such as cGAS trafficking between nuclear, cytoplasmic, and micronuclear compartments, also modulate the amplitude and type of immune response [50]. Overall, cytoplasmic DNA surveillance involves partially redundant sensors with overlapping yet distinct DNA‐binding preferences. Tight regulation of these pathways is essential to balance immune activation with genome integrity. In the context of persistent DNA damage, this balance can be disrupted, shifting immune responses toward chronic inflammation and senescence‐associated secretory phenotypes (SASPs).

### 3.3 DNA Damage‐Induced Organelle Stress Responses

Persistent nuclear DNA damage and cytoplasmic nucleic acid also perturb the structure and function of key cytoplasmic organelles (Figure 1.7, 9). Stress responses involving the ER, Golgi apparatus, and lysosomes are common consequences of genomic instability and contribute to the amplification of inflammatory signaling while impinge on cell fate decisions. Nuclear DNA damage indirectly disrupts ER homeostasis through several converging mechanisms. For instance, ROS produced downstream of the DDR contribute to protein misfolding, while transcriptional and translational errors associated with genotoxic stress increase the burden of unfolded or aberrant proteins in the ER [8, 51]. This imbalance activates the unfolded protein response (UPR), a conserved signaling network mediated by PERK, IRE1α, and ATF6. The UPR attempts to restore proteostasis by attenuating translation and promoting chaperone activity and degradation pathways [52]. Although direct DDR‐mediated activation of UPR sensors remains to be seen, morphological changes in the ER, including dilation and expansion, are consistently observed following DNA damage. This includes cells treated with topoisomerase inhibitors, doxorubicin, or methylmethane sulfonate [53, 54, 55]. Persistent ER stress sensitizes cells to DNA damage‐induced apoptosis and impairs energy metabolism. Notably, it suppresses glucose transporter 1 (GLUT‐1) and tricarboxylic acid (TCA) cycle activity, particularly in hematopoietic systems [56, 57]. Furthermore, cGAS–STING signaling, initiated by cytoplasmic DNA and localized to the ER membrane, provides a direct mechanistic link between nuclear DNA damage sensing and ER‐associated inflammatory output [58].

Likewise, the Golgi apparatus that modifies, sorts, and packages proteins and lipids for secretion or delivery is sensitive to genotoxic stress. Activation of the DNA damage sensor DNA‐PK leads to phosphorylation of GOLPH3, a Golgi membrane protein that interacts with MYO18A and F‐actin [59]. This phosphorylation enhances cytoskeletal tension on the Golgi, promoting its fragmentation and dispersion. Dilated ER and Golgi dispersal are also evident in macrophages treated with the DNA crosslinking agent mitomycin C and in *Ercc1* ^−^/^−^ macrophages deficient in multiple DNA‐repair pathways [54]. Notably, inhibition of ATM kinase reverses these changes, indicating that the cytoplasmic manifestations of nuclear DNA damage are both DDR‐dependent and reversible [54]. These observations suggest that ER enlargement and Golgi membrane expansion are adaptive responses. They enable cells to mitigate ER stress and recalibrate secretory capacity through membrane remodeling [60].

Lysosomes, the membrane‐bound organelles involved in cellular waste disposal, are also affected downstream of persistent DDR signaling. DNA damage induces lysosomal membrane permeabilization, allowing hydrolases such as cathepsins B and D to escape into the cytoplasm [61]. Although autophagy, the process of cleaning out damaged cells and recycling their components, is initially upregulated in response to DNA damage as a cytoprotective mechanism [62], the concurrent destabilization of lysosomes undermines this process. The leaked cathepsins can activate inflammatory pathways, including the AIM2 and NLRP3 inflammasomes, which further amplify cytokine production and cellular stress responses [63, 64]. Thus, lysosomal destabilization contributes to a feedback loop in which nuclear damage promotes cytoplasmic inflammation through organelle dysfunction. Together, these organelle‐specific responses amplify inflammation and reshape the secretory landscape characteristic of senescent and aging cells.

## 4 DNA Damage‐Induced Secretory Programs and Intercellular Communication

### 4.1 DAMPs as Early Mediators of Non‐Cell‐Autonomous Stress Signaling

Cytoplasmic DNA sensing initiates intracellular innate immune responses. In parallel, persistent genomic instability promotes the release of DAMPs that extend stress signals into the tissue environment (Figure 2.1, 2). Acting through autocrine and paracrine mechanisms, DAMPs coordinate short‐range communication and contribute to the early stages of tissue‐level dysfunction. Among typical DAMPs, HMGB1 is notable for its dual functionality. This nuclear protein is released either passively from necrotic cells or actively by stressed or senescent cells. In the extracellular space, HMGB1 binds to receptors such as RAGE and TLR4, triggering NF‐κB activation, cytokine production, and immune cell recruitment [65, 66] (Figure 2.2, 4). Its inflammatory activity is regulated by redox state. The disulfide form promotes cytokine production, whereas fully reduced HMGB1 acts as a chemokine, and the oxidized form is inactive [67]. This redox‐dependent variability complicates therapeutic targeting, as HMGB1 function depends on cellular context and stress intensity.

Extracellular ATP, another major DAMP, is released by viable but stressed cells through pannexin‐1 channels or lysosomal secretion. It activates the NLRP3 inflammasome through P2 × 7 receptors, leading to IL‐1β and IL‐18 secretion [68, 69] (Figure 2.5). ATP also engages P2Y2 receptors to recruit neutrophils and enhance cytokine output, particularly in inflamed or senescent tissues [70]. Although well‐characterized in immune cells, ATP signaling effects in epithelial or stromal compartments remain underexplored, particularly under chronic DNA damage, where ATP may act as a context‐dependent amplifier or suppressor of inflammation.

Heat shock proteins (HSPs) (HSP70, HSP90), classically described as intracellular chaperones, also act extracellularly as DAMPs in response to DNA damage [71]. When secreted, they interact with receptors such as TLR2, TLR4, and CD91, enhancing antigen presentation and inflammatory activation [72, 73]. Although extracellular HSPs are classically proinflammatory, they may also promote immunosuppressive signaling. This dual role is particularly evident in cancer and tissue repair contexts and remains incompletely understood.

In addition to protein‐based DAMPs, noncoding RNAs have been implicated in paracrine immune signaling. Telomeric repeat‐containing RNA (TERRA), typically localized to chromosome ends, accumulates in the cytoplasm under telomeric stress, particularly in cells with impaired checkpoint control. There, it binds to ZBP1 and activates MAVS signaling at the mitochondrial membrane, inducing Type I interferon responses independently of cGAS–STING or RIG‐I signaling [44, 74] (Figure 2.6). This suggests that under specific conditions, TERRA can contribute to innate immune activation. Whether this pathway functions in noncrisis contexts such as aging or chronic stress remains an open question.

### 4.2 SASP as a Driver of Inflammation and Tissue Dysfunction

Persistent DNA damage drives the development of the SASP, a broad secretory program characteristic of senescent cells (Figure 2.7). Although these cells are permanently growth‐arrested, they remain metabolically active and secrete a complex mixture of proinflammatory cytokines (e.g., IL‐6, IL‐8), chemokines (e.g., CXCL1), growth factors, and matrix‐remodeling enzymes such as matrix metalloproteinases (MMPs) (Figure 2b) [75]. SASP factors establish a proinflammatory microenvironment that reinforces senescence in neighboring cells, a process known as the bystander effect [76].

SASP development is transcriptionally regulated by persistent DDR signaling, activation of the cGAS‐STING pathway, and downstream effectors such as NF‐κB and C/EBPβ [77, 78]. Mitochondrial dysfunction, often marked by increased ROS production, further amplifies SASP expression by sustaining innate immune activation [79] (Figure 2.8). Notably, SASP composition is dynamic and context‐dependent, influenced by cell type, tissue environment, and the nature of the initiating genomic insult [80, 81]. Extracellular matrix components and mechanical stress inputs can further modify SASP profiles, underscoring the role of tissue context in shaping secretory outcomes [82].

Although transient SASP activity may promote tissue repair and immune clearance of damaged cells, its persistence under unresolved genomic instability has been associated with fibrosis, stem cell exhaustion, and local tissue degeneration [75]. Recent studies also demonstrate that SASP factors can be packaged into EVs, transmitting prosenescent signals via protein and RNA cargo [83]. The spatial extent and coordination of SASP signaling remain active areas of investigation. These findings raise important questions about whether SASP represents an adaptive response or a secondary consequence of damage accumulation.

Beyond immune activation, SASP factors affect a range of nonimmune cells, including epithelial, stromal, and neuronal lineages. These signals can disrupt extracellular matrix organization, impair barrier integrity, and alter progenitor cell behavior, contributing to tissue dysfunction [84]. Whether these effects are adaptive or pathogenic likely depends on the dosage, duration, and context of SASP exposure. It remains unresolved whether SASP secretion is a passive consequence of damage or an actively regulated program evolved for tissue‐level adaptation. Recent findings suggest that SASP composition and delivery are subject to regulatory controls, including vesicular trafficking and stress signaling pathways [85, 86]. Elucidating these control mechanisms may offer new therapeutic strategies to selectively suppress deleterious outputs while preserving beneficial aspects of the stress response.

### 4.3 Extracellular Vesicles as Systemic Mediators of Damage‐Induced Signaling

Beyond autocrine and paracrine loops, persistent DNA damage also activates systemic responses that affect distant tissues. These endocrine‐like signals are mediated by EVs, circulating cytokines, and metabolic regulators. Initially, they may support damage containment and stress adaptation. However, their chronic activation contributes to age‐associated tissue degeneration, immunosenescence, and metabolic dysfunction [1, 87].

A central mechanism for long‐range stress signaling is the release of EVs, including exosomes and microvesicles, from DNA‐damaged or senescent cells. These vesicles act as endocrine messengers, carrying DNA fragments, oxidized nucleic acids, noncoding RNAs (miRNAs, lncRNAs), and damage‐associated proteins that travel through the circulation (Figure 2.9). In recipient cells, especially in immune and endothelial tissues, DNA‐containing EVs from irradiated or aged sources activate the cGAS–STING pathway, inducing interferon responses and IL‐6 secretion [88]. This activation contributes to chronic low‐grade inflammation and vascular dysfunction. The downstream effects of cGAS–STING activation vary by context, driving antiviral signaling in immune cells and promoting senescence or matrix remodeling in epithelial tissues.

Under genotoxic stress, EV cargo becomes selectively enriched with immunostimulatory components. For example, oxidized mitochondrial DNA and RNA:DNA hybrids are preferentially loaded into vesicles [83, 89]. However, the mechanisms underlying DNA packaging into immunogenic vesicles remain unclear. Possible routes include ESCRT‐dependent sorting, autophagy, and nuclear envelope rupture, each potentially generating vesicles with distinct immunological profiles [90].

In addition to DNA cargo, senescence‐associated EVs carry regulatory RNAs that reshape metabolic and regenerative pathways in recipient cells. Circulating miRNAs such as miR‐146a, miR‐34a, and miR‐21, enriched in EVs from DNA‐damaged or senescent cells, suppress IGF‐1R signaling, disrupt mitochondrial function, and impair insulin sensitivity in metabolic tissues like liver, adipose, and muscle [91]. This establishes a mechanistic link between localized genomic instability and systemic metabolic aging. In line with this, we recently showed that EVs derived from DNA repair‐deficient macrophages reprogram metabolic activity in recipient cells, promoting insulin‐independent glucose uptake, enhanced oxidative phosphorylation, and systemic metabolic remodeling in vivo, which are hallmarks of inflammation and aging [54]. Similarly, EVs from senescent bone marrow macrophages induce senescence and inflammatory gene expression in mesenchymal stem cells, impairing hematopoietic niche function and contributing to immunosenescence [92]. Interestingly, under certain conditions, EVs may support regenerative outcomes, particularly in premalignant or regenerative niches, although the mechanisms governing these context‐dependent effects are not fully understood [93].

Together, these findings highlight EVs as key mediators of systemic communication during persistent DNA damage. By carrying nucleic acids and regulatory signals across tissue boundaries, EVs not only reinforce local inflammatory and stress responses but also promote dysfunction in distant cells and tissues. These non‐cell‐autonomous mechanisms link localized genome instability to widespread tissue degeneration and organismal aging. Understanding how EV cargo, trafficking, and target cell responses are regulated under chronic genomic stress will be essential for understanding and modulating these systemic effects.

## 5 Consequences of Systemic DDR Signaling

**FIGURE 3:**
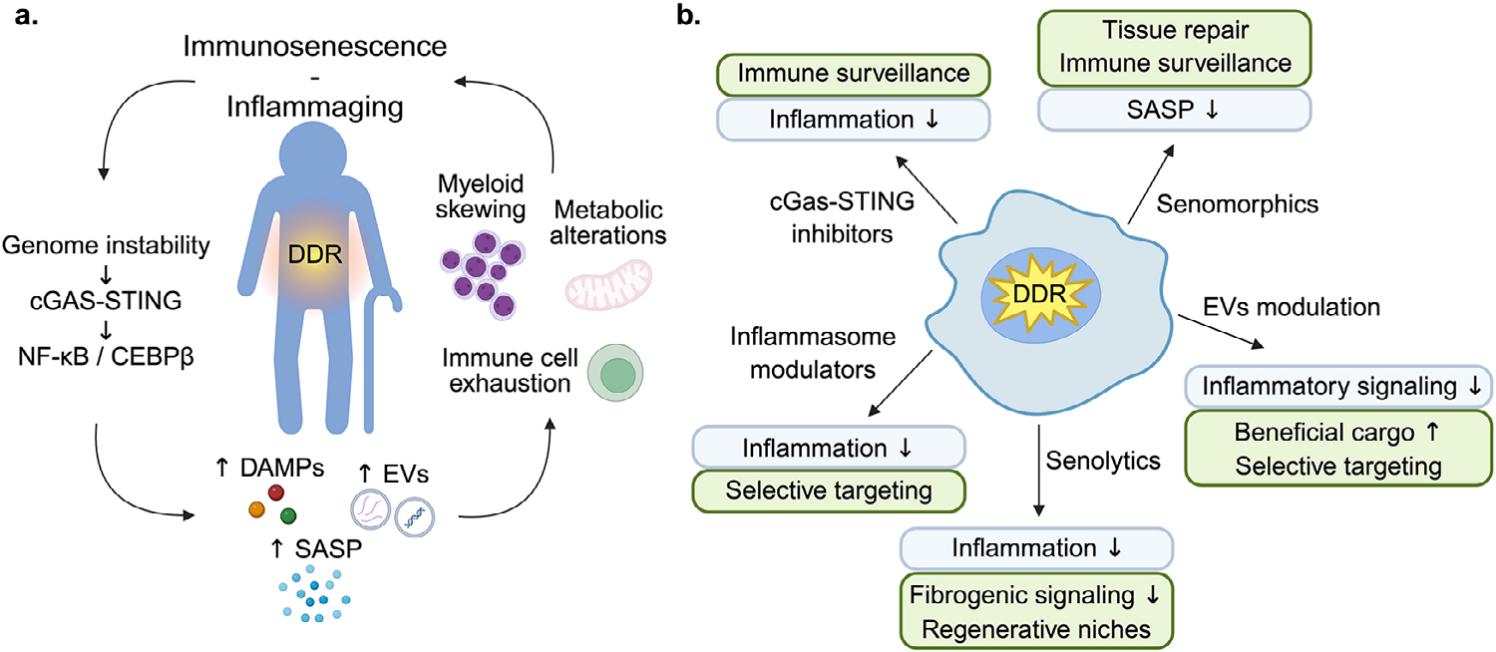
Systemic consequences of persistent DNA damage and potential therapeutic interventions. (a) Persistent DNA damage response (DDR) and chronic genome instability drive age‐related immunosenescence and inflammaging. This occurs through the activation of cGAS‐STING and NF‐κB–C/EBPβ signaling, which elevates the release of damage‐associated molecular patterns (DAMPs), senescence‐associated secretory phenotype (SASP) factors, and extracellular vesicles (EVs). These secreted mediators, along with resulting myeloid skewing, metabolic alterations, and immune cell exhaustion, fuel the decline of immune function and chronic inflammation. (b) To mitigate these detrimental systemic effects, various therapeutic strategies are under investigation. These include cGAS‐STING inhibitors and inflammasome modulators designed to reduce excessive inflammation, ideally while preserving immune surveillance and achieving selective targeting. Senolytics aim to eliminate senescent cells, thereby decreasing inflammatory and fibrogenic signals, though effects on regenerative niches require careful consideration. Senomorphics seek to modulate SASP composition, reducing its harmful aspects while retaining beneficial effects on tissue repair and immune functions. Furthermore, EV modulation focuses on reducing proinflammatory EV release or engineering EVs with beneficial cargo, while addressing challenges like off‐target effects. The uncertainties in these approaches underscore the complexity of balancing therapeutic efficacy with the preservation of essential physiological processes. Created in BioRender. Siametis, A. (2025) https://BioRender.com/n50ybfh.

Chronic genome instability sustains the release of damage‐associated cytokines, EVs, and metabolic regulators that impair tissue function and systemic homeostasis (Figure 3a). Although initially protective, these systemic responses extend beyond the local DDR, contributing to aging phenotypes and functional decline [8, 94, 95]. A key systemic change involves a reduction in circulating anabolic signals, such as insulin‐like growth factor 1 (IGF‐1), fibroblast growth factors (FGFs), and adipokines, alongside a metabolic shift favoring stress resistance over growth [96]. Although transient suppression of IGF‐1 signaling enhances survival during acute genotoxic stress, chronic reduction promotes endocrine aging, frailty, and metabolic rigidity [97, 98]. It remains unclear whether these hormonal shifts are passive consequences of tissue dysfunction or actively orchestrated through DDR‐regulated transcription factors such as p53, FOXO, and NRF2.

Circulating inflammatory cytokines, including IL‐6, IFN‐β, and TNF‐α, are induced downstream of DNA sensing and SASP activation. These cytokines can cross tissue boundaries, including the blood–brain barrier, and reprogram distant microenvironments, including hematopoietic niches, where they promote myeloid skewing, a feature of immunosenescence [99, 100]. Alongside cytokines, EVs disseminate DNA fragments, miRNAs, and damage‐associated proteins to distant tissues. These vesicles can reinforce a feedforward loop of senescence and inflammation in otherwise undamaged tissues [30, 92].

The immune system, serving as both a sensor and effector of damage signals, is particularly vulnerable to persistent damage signaling. Chronic activation of the cGAS‐STING pathway and the ATM‐NEMO‐NF‐κB axis leads to sustained interferon signaling and cytokine production. This inflammatory state depletes lymphoid precursors, impairs T cell function, and promotes the expansion of senescent or exhausted immune populations [101]. This state of “inflammaging” compromises immunity and accelerates age‐related pathologies, including cardiovascular and neurodegeneration disorders. In the nervous system, senescent glial cells amplify neuroinflammation, linking immune remodeling to cognitive decline [102]. Notably, while prolonged STING signaling is detrimental, transient activation may support tissue regeneration, highlighting its context‐dependent effects [103].

Tissue regenerative capacity is similarly impaired by EVs from senescent or DNA‐damaged cells, which impair stem and progenitor cell function in niches, such as bone marrow and skeletal muscle [92]. Mesenchymal stem cells chronically exposed to damage signals show reduced proliferation, increased inflammatory gene expression, and reduced lineage plasticity [104, 105]. Remarkably, NF‐κB‐driven SASP activity can be maintained independently of DDR signaling through GATA4, underscoring the self‐perpetuating nature of these stress programs [106].

Metabolically active tissues are especially vulnerable. EVs from DNA repair‐deficient macrophages have been shown to reprogram metabolism in target tissues, promoting insulin‐independent glucose uptake and low‐grade inflammation [54]. Conversely, EVs from mesenchymal stem cells can restore mitochondrial function and proteostasis in aged tissues, highlighting the dual potential of EV‐mediated communication depending on context [107]. Whether the outcome is protective or detrimental likely depends on both vesicle origin and the physiological state of the recipient tissue.

DNA damage‐associated transcription stress introduces additional systemic challenges [108]. Unresolved transcriptional blocks and RNA:DNA hybrid processing generate cytoplasmic nucleic acid fragments that activate innate immune sensors such as cGAS–STING [30]. Damaged transcripts and aberrant proteins impose proteotoxic stress on postmitotic tissues like brain and muscle, overloading chaperone systems and activating the UPR [109]. These proteotoxic and inflammatory feedback loops perpetuate tissue degeneration even after the initiating genotoxic insult has resolved.

Collectively, these disruptions reveal that aging arises not from isolated cellular damage but from a breakdown in systemic coordination under chronic genotoxic stress [110]. Inflammation accelerates further damage accumulation; impaired stem cell function constrains repair; endocrine suppression limits metabolic resilience; and proteostasis failure undermines tissue integrity. Persistent DNA damage thus drives tissue degeneration through cell loss as well as by disrupting intercellular communication.

## 6 Targeting Secretory Pathways of DNA Damage

### 6.1 Targeting Inflammatory Mediators in DNA Damage‐Induced Secretion

Chronic cGAS‐STING pathway activation, driven by unresolved DNA damage, contributes to stem cell dysfunction, metabolic decline, and tissue inflammation during aging. To mitigate these effects, several small‐molecule inhibitors of cGAS (e.g., RU.521, PF‐06928125) and STING (e.g., H‐151, C‐176) have shown promise in attenuating Type I interferon signaling without broadly suppressing immune vigilance [111, 112]. Such targeted inhibition may be especially useful in aging contexts, where maintaining immune surveillance while minimizing collateral inflammation is critical. Spatial regulators of cGAMP metabolism represent an additional therapeutic target. ENPP1, an ectonucleotidase that degrades extracellular cGAMP, and transporters such as SLC19A1, which mediate cGAMP uptake, modulate the spread of inflammatory signaling beyond the site of damage [112, 113]. These regulators may enable compartmentalized control of inflammation, reducing systemic spillover from localized DNA damage.

The context‐dependent nature of STING signaling is increasingly recognized. Although prolonged activation drives dysfunction, short‐term STING signaling can promote tissue repair and immune clearance of senescent cells [114, 115]. These observations point to temporally controlled therapeutic strategies, such as pulsatile dosing or inducible inhibitors, that preserve beneficial outputs while minimizing chronic activation. Whether these kinetics can be manipulated clinically remains an open but promising area of investigation.

Therapeutic modulation of inflammasome activity offers another promising strategy to mitigate chronic inflammation. NLRP3 inhibitors such as MCC950 have shown efficacy in preclinical models of age‐related inflammation, including neurodegenerative disorders [116, 117]. As with interferon tuning, the therapeutic goal is not complete suppression but modulation of excessive, chronic activity. However, selective targeting remains challenging, particularly for inflammasomes like AIM2, where therapeutic tools are limited and functional redundancy complicates intervention [118].

Targeting the SASP is another promising strategy for mitigating DNA damage‐induced inflammation. Several compounds have been identified that modulate SASP output without reversing growth arrest. These include rapamycin (an mTOR inhibitor), ruxolitinib (a JAK–STAT pathway inhibitor), and GATA4–NF‐κB axis blockers [119]. These compounds suppress the SASP, reduce frailty indices, and delay functional decline in rodents [120]. Rapamycin also decreases progerin accumulation in Hutchinson‐Gilford progeria syndrome (HGPS) fibroblasts [121]. Clinically, the farnesyltransferase inhibitor lonafarnib, originally developed to blunt progerin prenylation, improves weight gain, arterial stiffness, and lifespan in HGPS children and now stands as the first FDA‐approved therapy for a progeroid disorder [122]. Although these senomorphic agents reduce proinflammatory secretory burden, a key challenge lies in preserving beneficial components of the SASP, those involved in tissue repair, immune recruitment, or clearance of damaged cells. Broad SASP suppression may blunt these adaptive functions. Thus, the field is moving toward strategies that discriminate between degenerative and regenerative outputs. Achieving this will require a refined understanding of the upstream cues, such as lesion type, epigenetic landscape, and transcriptional context, that dynamically shape SASP composition in vivo.

Beyond targeting inflammation or SASP, there is growing interest in modulating the DDR itself as a therapeutic strategy. Small‐molecule inhibitors of key DNA repair proteins (e.g., PARP, ATM) are already in clinical use, primarily in oncology. Recent work has underscored that these agents can influence systemic homeostasis beyond their intended cytotoxic effects. For example, PARP inhibitors, while effective at exploiting vulnerabilities in tumor cells, can also promote the accumulation of DNA breaks via PARP trapping, leading to increased cytosolic DNA and activation of innate immune sensors, including the cGAS‐STING pathway [123]. Similarly, ATM inhibitors have been shown to trigger mitochondrial DNA leakage into the cytosol, further stimulating innate immunity and potentially enhancing antitumor responses [124]. These findings highlight the complex interplay between genome maintenance and immune signaling, raising important questions about repurposing DNA repair‐targeting drugs to address age‐related inflammation and tissue dysfunction. Although such approaches hold potential, careful evaluation of long‐term toxicity and the risk of exacerbating genomic instability will be essential.

### 6.2 Modulating Extracellular Vesicles to Intercept Damage‐Associated Signaling

EVs are emerging as key mediators of non‐cell‐autonomous responses to DNA damage. Therapeutic interventions target two main pathways: inhibition of pathogenic EV release and engineering of reparative EVs. Inhibitors such as GW4869, which block neutral sphingomyelinase 2 (nSMase2), reduce exosome biogenesis and inflammatory EV signaling in aging models [125, 126]. Limiting vesicle output may be particularly valuable in fibrotic or chronic inflammatory conditions, where damage signals persist. In contrast, EVs derived from healthy or reprogrammed cells exhibit therapeutic potential. These vesicles often contain miR‐21, miR‐146a, NAMPT, and SOD2, which support redox balance, autophagy, and mitochondrial function in recipient cells [127, 128]. EVs enriched for extracellular nicotinamide phosphoribosyltransferase (eNAMPT) restore mitochondrial function, enhance NAD⁺ levels, and attenuate tissue decline in vivo [129]. Therapeutic benefits have been shown in models of cardiovascular aging, metabolic dysfunction, and neurodegeneration [107, 130, 131]. To harness these effects, several approaches for EV optimization are under development. Ex vivo engineering of donor cells allows the enrichment of desired vesicle cargo, while preconditioning cells with caloric restriction or hypoxia enhances the antiinflammatory properties of released EVs [132, 133, 134, 135]. Surface tagging of EVs with tissue‐specific ligands may further improve targeting precision and reduce off‐target effects. Recent advances in single‐EV omics now allow detailed characterization of EV subtypes by cargo, size, and cellular origin [136]. These techniques hold promise for both diagnostic applications and the precision design of therapeutic vesicles. By distinguishing vesicle subpopulations associated with degenerative signaling from those promoting repair, it may become feasible to selectively modulate EV pathways in a tissue‐ and context‐specific manner.

### 6.3 Targeting DNA Damage‐Induced Homeostatic Imbalance

Persistent DNA damage disrupts mitochondrial metabolism, NAD⁺ balance, and immune composition, perturbing cellular homeostasis and reinforcing maladaptive secretory phenotypes [6, 137]. Interventions that address these internal dysfunctions may indirectly reshape the extracellular signaling environment and reduce the burden of chronic inflammation.

Senotherapeutics have emerged as one such strategy. Senolytics, such as dasatinib combined with quercetin, selectively eliminate senescent cells and extend median lifespan, reverse metabolic dysfunction, and enhance physical performance in murine models [138]. Early human trials confirm senescent‐cell clearance and safety in patients with diabetic kidney disease [139] and mild Alzheimer's disease [140], while in Werner syndrome models, quercetin lowers the senescent‐cell burden [141]. However, concerns remain about their specificity and potential impact on regenerative niches. Senomorphics, like metformin or resveratrol, offer a more subtle alternative by suppressing SASP output while maintaining cell viability [119]. These compounds modulate key pathways such as AMPK, SIRT1, and mTOR, supporting mitochondrial function and oxidative homeostasis.

Rejuvenating mitochondrial function is another key strategy. NAD^+^ precursors such as nicotinamide riboside (NR) and nicotinamide mononucleotide (NMN) enhance oxidative metabolism and DNA repair [142, 143]. Meanwhile, Urolithin A and other mitophagy activators facilitate clearance of dysfunctional mitochondria, limiting the release of mitochondrial DNA into the cytoplasm [144]. Together, these interventions may help reduce intracellular sources of damage‐associated signals.

Immune modulation complements these strategies. Chronic DNA damage drives T‐cell exhaustion, macrophage skewing toward proinflammatory phenotypes, and myeloid bias in hematopoiesis [145]. Approaches such as low‐dose checkpoint inhibition, administration of antiinflammatory cytokines (e.g., IL‐10), and dietary interventions (e.g., fasting mimetics) are being explored to restore immune balance, preserve surveillance, and prevent the spread of senescence. However, immune responses are highly context‐dependent, shaped by age, tissue type, and sex. For example, sex‐specific responses to IL‐6 and Type I interferons have been documented in aging models, emphasizing the importance of tailoring interventions to individual physiological states [146]. Together, these strategies aim to reverse internal dysfunctions induced by persistent DNA damage, modulate the secretory phenotype, and restore homeostasis in the aging cellular environment.

## 7 Concluding Remarks

The traditional view of DNA damage as a localized, cell‐intrinsic lesion requiring repair is rapidly giving way to a broader paradigm: one in which genotoxic stress acts as a powerful initiator of systemic signaling that reshapes organismal physiology. Persistent genomic instability engages cytoplasmic sensing pathways and orchestrates secretory programs that deploy DAMPs, SASP factors, and EVs to propagate stress signals across cellular and tissue boundaries. These non‐cell‐autonomous responses are not passive byproducts of damage, but active processes that recalibrate tissue homeostasis. Although acutely beneficial, mobilizing immune responses and promoting regeneration, their chronic activation drives key hallmarks of aging, including inflammaging, metabolic dysfunction, stem cell attrition, and loss of tissue coordination. This evolving perspective reframes aging not merely as the accumulation of damage, but as a progressive failure of intercellular communication networks triggered by unresolved DNA lesions. Recognizing DNA damage as a systemic communicator, not simply as a molecular defect, opens transformative therapeutic possibilities. Beyond repairing DNA itself, emerging strategies aim to modulate the downstream signaling it initiates. These include targeting master regulators such as STING and inflammasomes, reprogramming the SASP to favor regeneration over degeneration, engineering or intercepting EV‐mediated signaling, and restoring cellular equilibrium (Figure 3b). The therapeutic vision has shifted from indiscriminately silencing damage responses to editing them–preserving beneficial outputs while neutralizing those that drive decline. Yet, this therapeutic promise is tempered by complexity. The outcomes of damage‐induced signaling are profoundly context‐dependent, shaped by tissue identity, age, sex, metabolic state, and immune tone. Future progress depends on decoding these contextual layers and developing precision interventions capable of rewiring communication networks with temporal, spatial, and molecular specificity. By shifting the focus from damage to dialogue, from genomic instability to its systemic echoes, we move closer to a new biology of aging. One in which resilience is not merely restored by fixing what breaks, but by reestablishing the cellular conversations that sustain coordinated function across the lifespan.

## Author Contributions

**AS** and **GAG** wrote the manscript.

## Conflicts of Interest

The authors declare no conflicts of interest.

## Data Availability

Data sharing is not applicable to this article, as no new data were created or analyzed in this study.
